# Human Milk Virome Analysis: Changing Pattern Regarding Mode of Delivery, Birth Weight, and Lactational Stage

**DOI:** 10.3390/nu13061779

**Published:** 2021-05-23

**Authors:** Meltem Dinleyici, Vicente Pérez-Brocal, Sertac Arslanoglu, Ozge Aydemir, Sibel Sevuk Ozumut, Neslihan Tekin, Yvan Vandenplas, Andrés Moya, Ener Cagri Dinleyici

**Affiliations:** 1 Department of Social Pediatrics, Faculty of Medicine, Eskisehir Osmangazi University, Eskisehir 26040, Turkey; meltemayata@hotmail.com; 2 Genomics and Health Area, Foundation for the Promotion of Sanitary and Biomedical Research of Valencia Region (FISABIO-Public Health), 46020 Valencia, Spain; perez_vicbro@gva.es (V.P.-B.); andres.moya@uv.es (A.M.); 3 CIBER in Epidemiology and Public Health (CIBEResp), 28029 Madrid, Spain; 4 Division of Neonatology, Faculty of Medicine, Medeniyet University, Istanbul 34720, Turkey; sertacarslanoglu@gmail.com (S.A.); sibel.ozumut@gmail.com (S.S.O.); 5 Division of Neonatology, Faculty of Medicine, Eskisehir Osmangazi University, Eskisehir 26040, Turkey; drozgegenc@yahoo.com.tr (O.A.); tekinneslihan@yahoo.com (N.T.); 6 Department of Pediatrics, KidZ Health Castle, UZ Brussel, Vrije Unversiteit Brussel, 1050 Brussels, Belgium; Yvan.Vandenplas@uzbrussel.be; 7 Institute for Integrative Systems Biology (I2SysBio), The University of Valencia and The Spanish National Research Council (CSIC-UVEG), 46010 Valencia, Spain; 8 Department of Pediatrics, Faculty of Medicine, Eskisehir Osmangazi University, Eskisehir 26040, Turkey

**Keywords:** human milk, breastfeeding, viruses, microbiome, delivery mode

## Abstract

The human milk (HM) microbiota is a significant source of microbes that colonize the infant gut early in life. The aim of this study was to compare transient and mature HM virome compositions, and also possible changes related to the mode of delivery, gestational age, and weight for gestational age. Overall, in the 81 samples analyzed in this study, reads matching bacteriophages accounted for 79.5% (mainly *Podoviridae, Myoviridae, and Siphoviridae*) of the reads, far more abundant than those classified as eukaryotic viruses (20.5%, mainly *Herpesviridae*). In the whole study group of transient human milk, the most abundant families were *Podoviridae* and *Myoviridae*. In mature human milk, *Podoviridae* decreased, and *Siphoviridae* became the most abundant family. Bacteriophages were predominant in transient HM samples (98.4% in the normal spontaneous vaginal delivery group, 92.1% in the premature group, 89.9% in the C-section group, and 68.3% in the large for gestational age group), except in the small for gestational age group (only ~45% bacteriophages in transient HM samples). Bacteriophages were also predominant in mature HM; however, they were lower in mature HM than in transient HM (71.7% in the normal spontaneous vaginal delivery group, 60.8% in the C-section group, 56% in the premature group, and 80.6% in the large for gestational age group). Bacteriophages still represented 45% of mature HM in the small for gestational age group. In the transient HM of the normal spontaneous vaginal delivery group, the most abundant family was *Podoviridae*; however, in mature HM, *Podoviridae* became less prominent than *Siphoviridae*. *Myoviridae* was predominant in both transient and mature HM in the premature group (all C-section), and *Podoviridae* was predominant in transient HM, while *Siphoviridae* and *Herpesviridae* were predominant in mature HM. In the small for gestational age group, the most abundant taxa in transient HM were the family *Herpesviridae* and a species of the genus *Roseolovirus*. Bacteriophages constituted the major component of the HM virome, and we showed changes regarding the lactation period, preterm birth, delivery mode, and birth weight. Early in life, the HM virome may influence the composition of an infant’s gut microbiome, which could have short- and long-term health implications. Further longitudinal mother–newborn pair studies are required to understand the effects of these variations on the composition of the HM and the infant gut virome.

## 1. Introduction

Breastfeeding is considered a gold standard in infant nutrition, as it has been adapted to provide for the demanding growth of the newborn in a time-dependent way with a wide range of components including macronutrients (fat, proteins, and carbohydrates), micronutrients, and bioactives [1,2]. Breastfeeding has positive effects on infants from nutritional, physiological, and developmental viewpoints [1]. Breastfeeding boosts the immune system, enhances neurodevelopment, and can have an effect on the development of noncommunicable diseases and conditions later in life [1,3].

Human milk (HM) bioactives such as the HM microbiota have been shown to provide healthy gastrointestinal mucosal stimuli, influence the gut microbiota composition, and promote the development of the infant’s immune system [4,5]. The gut microbiota of an infant is formed during the first thousand days of life. Changes in the microbial composition during this period can affect health later in life [2]. The HM microbiota is a significant source of microbes that colonize the infant gut early in life, promote the growth of beneficial microbiota, aid in the maturation of the innate and adaptive immune systems, provide protection against gastrointestinal infections, and may help to form both short- and long-term infant health outcomes through metabolic programming, immunomodulation, and neuromodulation [4,5,6,7,8]. Breastfeeding affects the regulation of intestinal, neurological, and behavioral functions via the gut–brain axis in early life, which may imprint on the central nervous system and immune system throughout the lifespan. The HM microbiota is an infinite source for the infant gut microbiota as long as breastfeeding is maintained [1].

While the microbial communities are dominated by bacteria, viruses, archaea, and fungi may play a key role in maintaining gut homeostasis and are able to directly affect the host’s health [9]. While the early establishment and development of the HM and infant’s intestinal bacterial components of the microbiome have been well documented [10,11,12], knowledge about the acquisition of gut virome communities is limited [13,14]. Lim et al. [14] characterized changes in the gut virome and found that the gut bacteriophage population structure consisted primarily of a rich and diverse set of phages; richness decreased with age since birth. The first process of viral colonization in early life is characterized by the induction of prophages from pioneer bacteria, followed by colonization with viruses infecting human cells, the latter of which is modulated by breastfeeding [15]. Recent evidence suggests that breastfeeding affects the neonatal virome. HM viruses are transmitted from mother to infant through breastfeeding, and HM and infant stool viromes from a mother–infant dyad share substantial bacteriophage homology [16,17]. As a result, the virome transmitted by HM to the child could have a substantial effect on the infant’s long-term health [5].

The HM composition, as well as the microbiota composition, is influenced by maternal factors during pregnancy and lactation including geography, maternal diet and maternal psychosocial status, the mode of delivery and gestational age, intrapartum antibiotic use, lactation period (colostrum, transitional, and mature HM), and length of gestation [2,9,11]. Potential changes or alterations in the HM virome due to maternal/perinatal factors might play a role in defining the gut virome during the early life period, which could have long-term health implications. While most research has focused on the bacterial communities in HM, relatively little is known about the community of viruses in HM. This study aims to evaluate the HM virome composition with detailed metagenomic analysis, to compare transient and mature human milk, and also to evaluate possible changes related to preterm birth, delivery mode, and birth weight for gestational age. 

## 2. Material and Methods

This is a prospective study which was performed in two university hospitals in Turkey. All women delivered in the two hospitals where they were assessed after birth as part of the study protocol. The study’s design included five separate groups. Normal spontaneous vaginal delivery at term or C-section (emergency or elective) at term was the mode of delivery. Prematurity was described as a gestational age of less than 37 weeks [18], and premature infants aged less than 37 weeks but more than 32 weeks were included in the study. According to the InterGrowth-21st map [19], small for gestational age (SGA) newborns have a birth weight (BW) below the 10th percentile and high for gestational age (LGA) newborns have a BW above the 90th percentile for gestational age. 

Multiple pregnancies, maternal age <18 years or >45 years, maternal BMI > 30 kg/m^2^, use of antibiotics and/or probiotics during pregnancy or lactation, intrapartum antibiotic prophylaxis, and mothers with gastrointestinal system disorder or psychiatric disorders were all criteria for exclusion from the study group. The following information was collected: maternal age, parity, mode of delivery, gestational age, birth weight, and infant gender. 

Human milk collection and storage: All mothers gave their informed consent, which included signing a consent form authorizing the collection of milk samples and subsequent examination. HM samples were obtained from mothers recruited from two university hospitals in Turkey at two separate times: transient human milk samples (TMS; postpartum 7–15 days) and mature human milk samples (MMS; postpartum 45–90 days). All HM samples were foremilk and were collected in the morning at the hospital. Mothers were asked to clean their nipples and surrounding areas with sterile saline before collecting 3–5 mL of HM in sterile tubes. Hand expression was used to collect all HM samples. Until DNA extraction, all samples were held at −20 °C.

DNA extraction: DNA extraction was performed with QuickGene DNA tissue kit S (DT-S; Kurabo, Osaka, Japan) according to the manufacturer’s protocol. After first thawing on ice, the 1.5 mL milk sample was transferred to a 2 mL microtube. It was centrifuged at 5000 g for 7 min. Fat and the supernatant component of the milk were carefully removed. An MDT solution of 180 µL and a 25 µL EDT solution were added onto the pellet, and the pellet was left to incubate at 56 °C for 15 min after pipetting until dispersed. An LDT solution of 180 µL was added to the microtube and vortexed for 15 s. After that, the microtube was left to incubate at 70 °C for 10 min. In the next step, 240 µL of 99% cold ethanol was added and vortexed for 15 s. All the contents of the microtube were transferred to the QuickGene (Kurabo, Osaka, Japan) filter cassette, and the washing and elution process was performed following the instrument’s protocol. Washing was performed three times using a 750 µL washing solution. At the end of the extraction process, an average of 30–40 ng of genomic DNA was obtained, diluted with 50 µL of elution. Extracted DNA concentrations were measured using a Qubit 3.0 Fluorometer (Thermo Fisher Scientific; Life Technologies, Carlsbad, CA, USA), shotgun libraries were prepared from 1 ng of each sample using the Nextera XT DNA Library Prep Kit (Illumina, San Diego, CA, USA) following the manufacturer’s instructions, and paired-end sequencing was carried out in the NextSeq 500 sequencer (Illumina, San Diego, CA, USA).

Sequencing data and statistical analysis. The resulting reads were trimmed and quality and length filtered with the PRINSEQ-lite program [20]. Paired reads were joined using the FLASH program [21] with default parameters, and human-origin reads were removed by mapping against the human genome database (GRCh38.p11, reference human genome, December 2013) using Bowtie 2 [22] with local and very sensitive options. Next, two further mapping steps against bacterial and fungal reference genome datasets (downloaded from the NCBI FTP site in August 2016 and January 2018, respectively) with local and very sensitive options were also carried out using Bowtie 2, in order to remove those reads from the analysis of the virome. Only those reads that did not map were used for the BLAST algorithm search, using a tBLASTx strategy (*e*-value < 10^−5^, with identities ≥50%, 60% and 70%, ≥65% of the read length) against a customized viral database (March 2018), consisting of 99% identity clusters of complete viral genomes from the EBI and NCBI sites, plus all available viral sequences from the International Nucleotide Sequence Database Collaboration. It also included prophages from the PHAge Search Tool [23]. The minimum identity cutoff used was 65% for amino acids because viral genomes are more variable than bacterial and eukaryotic genomes, which evolve and diverge faster, leading to relatively low similarities compared with those seen in other organisms. Taxonomy was added to the BLAST results, and a lowest common ancestor strategy was used to assign the taxonomy to the hits using R v3.1.0 [24] customized scripts. These were also used for counting the reads abundance and constructing a taxonomically assigned reads abundance matrix for all samples. This matrix was converted into the Biological Observation Matrix format using the QIIME pipeline v1.9.0 for composition, abundance, and diversity analyses [25]. The presence or absence of viral taxa in the samples was analyzed at different taxonomic levels to determine their prevalence. Data were presented as mean ± standard deviation or median (range) for samples according to the collection time and delivery group, or both. In addition, the abundance of different viral reads at the species and family levels was calculated for all samples and group combinations. The Shannon diversity index was used to estimate the diversity within samples by using 1000 replicates of randomly chosen subsets of 18 reads per sample. Boxplots were created using R v3.1.0 and compared using two-tailed t-tests. The diversity between samples was analyzed using Bray–Curtis dissimilarity obtained with the QIIME pipeline for principal coordinate analysis. The nonparametric statistical method for multivariate analysis of variance Adonis was used for comparisons between categories [26]. Other statistical analyses, such as the nonparametric Wilcoxon–Mann–Whitney test for pairwise comparisons, were conducted with R scripts, while Kruskal–Wallis tests were implemented by the QIIME group_significance.py script.

## 3. Results

A total of 88 HM samples were obtained from 44 mothers. The entire study group’s maternal age, mode of delivery, gestational age, birth weight, gender, and human milk sampling time are summarized in Table 1. In this study, viruses were detected in 81 of 88 samples. The number of reads between TMS and MMS was similar, as was the Shannon index between the groups at different sampling times.

**Figure 1:**
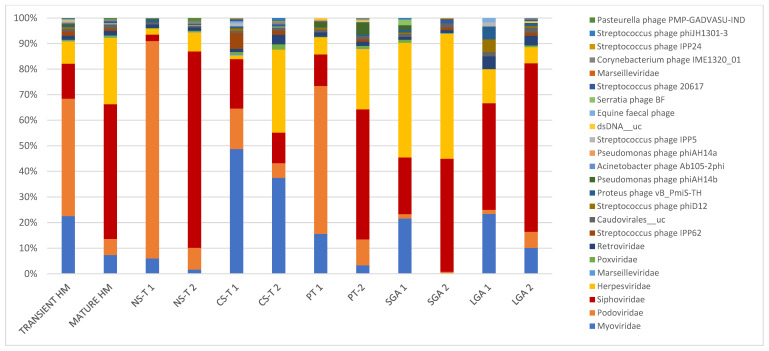
In transient and mature human milk samples, the most abundant taxa in terms of mode of delivery form, prematurity, and birth weight for gestational age. Bacteriophages were predominant in transient human milk samples: in the vaginal delivery group at 98.4%, at 92.1% in the premature group, at 89.9% in the C-section group, and at 68.3% in the LGA group, except in the SGA group (only ~45% bacteriophages in TMS). Bacteriophages were also predominant in mature human milk samples; however, they were lower in mature human milk samples than in transient human milk samples (71.7% in the vaginal delivery group, 60.8% in the C-section-term group, 56% in the premature group, and 80.6% in the LGA group). Bacteriophages accounted for 45% of mature human milk samples in the SGA group. NS-T: normal spontaneous vaginal delivery-term; CS-T: cesarean delivery-term; PT: premature; SGA: small for gestational age; LGA: large for gestational age for all groups; 1: transient milk; 2: mature milk.

Overall, in the 81 samples analyzed in this study, reads matching bacteriophages accounted for 79.5% (mainly *Podoviridae, Myoviridae, and Siphoviridae*) of the reads, far more abundant than those classified as eukaryotic viruses (20.5%, mainly *Herpesviridae*). The most abundant bacteriophage reads matched with members from the order *Caudovirales* (74.8%), particularly families *Siphoviridae* (34.6%), *Podoviridae* (24.6%), and *Myoviridae* (14.4%), with species *Staphylococcus phage St 134* (1.9%), *Streptococcus phage IPP62* (1.2%), *Staphylococcus phage Andhra* (1.2%), *Acinetobacter virus 133* (1.1%), *Staphylococcus virus SEP* (1%), *Enterococcus phage EFC-1* (0.91%), *Streptococcus phage YMC-2011* (0.85%), *Staphylococcus virus IPLAC1C* (0.7%), *Staphylococcus virus Sextaec* (0.67%), and *Clostridium phage vB_CpeS-CP51 18* (0.54%) being the most abundant ones. As for eukaryotic viruses, the most abundant order was *Herpesviridae* (18.0%), with *Human betaherpesvirus 5* (11.4%), *Human betaherpesvirus 6A* (0.63%), and *Human endogenous retrovirus K* (0.546%) as the most abundant species (Table 2, Figure 1).

Comparison of transient and mature milk samples.

While bacteriophages were predominant in TMS at 87.6%, they dropped to 67% in MMS. In TMS, the most abundant taxa were as follows: at the family level, *Podoviridae* (45.9%), *Myoviridae* (22.5%), *Siphoviridae* (13.7%), and *Herpesviridae* (8.8%); and at the species level, *Picovirinae_uc* (32.7%), *Sep1virus_uc* (13.6%), *Siphoviridae_n_n__uc* (4.6%), *Siphoviridae_n__uc* (4.2%), *Roseolovirus__uc* (4.2%), *Cp1virus__uc* (4.0%), *Staphylococcus phage St 134* (4.0%), and *Human betaherpesvirus 5* (3.3%). In MMS, the most abundant taxa were as follows: at the family level, *Siphoviridae* (52.6%), *Herpesviridae* (26.0%), and *Myoviridae* (7.4%), with *Podoviridae* decreasing to 6.8%; and at the species level, *Human betaherpesvirus 5* and *Siphoviridae_n__uc* (both 18.4%) (Table 2, Figure 1).

Comparison regarding delivery mode, gestational age, and birth weight for gestational age. 

Bacteriophages were predominant in transient HM samples: in the vaginal delivery group at 98.4%, at 92.1% in the premature group, at 89.9% in the C-section group, and at 68.3% in the LGA group, except in the SGA group (only ~45% bacteriophages in TMS). Bacteriophages were also predominant in mature HM samples; however, their appearance was lower than that in transient HM (71.7% in the vaginal delivery group, 60.8% in the C-section-term group, 56% in the premature group, and 80.6% in the LGA group). Bacteriophages, which were still lower than other groups, represented 45% of mature HM samples in the SGA group.

In the NS-T group, the most abundant taxa were as follows: at the family level, *Podoviridae* (84.9%) in TMS and *Siphoviridae* (76.7%) in MMS, with *Podoviridae* dropping to 8.5% in MMS; and at the species level, in TMS, *Picovirinae*_uc (55.5%), *Cp1virus_uc* (13.2%), and five bacteriophage strains, *Staphylococcus phage Andhra, Staphylococcus phage St 134, Halomonas phage phiHAP-1, Streptococcus virus Cp1,* and *Vibrio phage VP882*, and in MMS, *Siphoviridae*_n__uc and *Siphoviridae*_n_n__uc, accounting for half of the total viral reads (Table 2, Figure 1). While *Podoviridae* was the most abundant family in TMS in the NS-T group, this family accounted for 15.8% in the CS-T group, and *Myoviridae* was predominant (48.6%). In MMS of the CS-T group, *Myoviridae* was still the most abundant (37.4%), followed by *Herpesviridae* (32.5%), which accounted for only 1.5% of the reads in TMS of this group. *Sep1virus*__uc was the most abundant species in TMS (36.2%), and the second most abundant species (27.5%) in MMS of the CS-T group and the predominant species of MMS of the CS-T group was *Human betaherpesvirus 5* (30.0%). While approximately all premature newborns in the PT group were born via C-section, the most abundant taxa in the PT group in TMS and MMS were not similar to C-section-term newborns. In the PT group, the family *Podoviridae* (57.8%) was the most abundant in TMS, similar to the NS-T group, whereas *Siphoviridae* (53.2%) and *Herpesviridae* (24.7) were predominant in MMS. At the species level, the most abundant taxa were *Picovirinae*__uc in TMS (45.0%), similar to the NS-T group, and *Human betaherpesvirus 5* in MMS of the PT group (53.2%), similar to the CS-T group. In the SGA group, the most abundant taxa in TMS were *Herpesviridae* (44.8%) at the family level and *Roseolovirus*_uc (36.3%) and *Acinetobacter virus 133* (19.9%) at the species level, and in MMS, *Herpesviridae* (48.8%) at the family level and *Human betaherpesvirus 5* (27.6%) at the species level. In the LGA group, *Siphoviridae* was the predominant family in both TMS (41.6%) and MMS (65.8%), and *Siphoviridae*_n_n__uc was the most abundant species in both TMS (26.6%) and MMS (44.9%) (Table 2, Figure 1).

## 4. Discussion

This is a detailed metagenomic study of the HM virome in which we first demonstrated the impact of the delivery mode, prematurity, birth weight for gestational age, and lactation period (transient or mature milk) on the composition of HM viruses. We detected viruses (bacteriophages and eukaryotic viruses) in 92.0% of all HM samples, and, as in other HM microbiome studies, the virome composition was distinguished by certain interindividual variability; each mother harbored a morphologically distinct bacteriophage population [8]. Viromes have been found in the gut, skin, respiratory tract, blood, and cerebrospinal fluid. The gut virome is dominated by bacteriophages [9]. The HM virome, which contains eukaryotic viruses, bacteriophages, and viral elements incorporated in the host chromosomes, has been discovered to be distinct from adult stool and other body anatomical sites [5]. Human milk has been shown to be one of the earliest influences in the transmission of bacteriophages and eukaryotic viruses [27].

In this study, overall, the most abundant taxa were bacteriophages (79.5%; mainly *Podoviridae, Myoviridae, and Siphoviridae*), and the remaining taxa were eukaryotic viruses (20.5%, mainly *Herpesviridae*). While bacteriophages were predominant in TMS at 87.6%, they dropped to 67% in MMS. Bacteriophage predominance also varies according to preterm birth, mode of delivery, and birth weight for gestational age. Both pathogenic and non-pathogenic viruses (HIV, cytomegalovirus, Ebola, and Zika) can be transmitted to infants through HM [8,9,17,28]. Pannaraj et al. [16] analyzed the HM virome and found that bacteriophages (*Myoviridae, Siphoviridae,* and *Podoviridae* families) made up a substantial part (95%) of the viruses present in HM, with only a few eukaryotic viruses. Duranti et al. [17] analyzed HM and infant stool samples (postpartum 7 days and 1 month) and concluded that the bifidophage (*Bifidobacterium longum phage 10029*) was transmitted by the HM as part of the bifidobacterial host. Via their lytic and lysogenic cycles, HM bacteriophages can influence and modulate bacterial ecology [5]. Bacteriophages have the ability to destroy bacteria or provide them with potentially beneficial genes, thus forming the early life human microbiome [3,5,29]. Though HM bacteriophages have an effect on bacterial ecology in the infant gut, eukaryotic viruses can have a direct impact on infant health. The *Herpesviridae*, *Poxviridae*, *Mimiviridae*, and *Iridoviridae* families are the most common eukaryotic viruses [5]. The *Herpesviridae* family of eukaryotic viruses was found to be the most prevalent in our study, accounting for 8.8% of transient HM samples and 26% of mature HM samples.

There are some differences regarding the percentage of the bacteriophage composition in HM between our study and a previous study [9]. *Siphoviridae* and *Podoviridae* were the most abundant taxa in both studies. Pannaraj et al. [9] evaluated transient HM samples (4–10 days of infant age) of 10 healthy Hispanic mother–infant pairs (seven were vaginally delivered, four mothers received intrapartum antibiotics, and seven infants received HM and formula) in the United States. They showed that most viruses in HM (95.2%) were predicted to be bacteriophages. In our study, bacteriophages were also predominant in transient human milk samples (in the vaginal delivery group at 98.4%, at 92.1% in the premature group, and at 89.9% in the C-section group); however, they lower in the LGA group at 68.3% and in the SGA group at ~45%. Pannaraj et al. [9]. showed that the most abundant viruses in TMS were from the *Myoviridae* family. In our study, *Podoviridae* and *Myoviridae* were the most abundant families, similar to Pannaraj et al.’s study [9]. However, in MMS, *Podoviridae* became less abundant, with *Siphoviridae* being the most abundant family, followed by *Herpesviridae*. The percentage and distribution of the bacteriophage composition of Pannaraj et al. [9] are similar to our normal spontaneous vaginal delivery group; however, our lower rate in the whole study group might be related to the SGA group. The mother’s geographical location might also play a direct or indirect role in the HM microbiota composition. The influence of lifestyle, maternal diet, and environment on the composition of the HM microbiota has been suggested [2,8]. Recently, Maqsood et al. [30] found that cytomegalovirus (found in 98% of human milk samples) dominated the HM virome of HIV-positive mothers in Kenya, with the bacteriophage families *Myoviridae*, *Siphoviridae*, and *Podoviridae*; virome profiles and diversity were not substantially altered by HIV immunosuppression or associated with infant mortality.

In the present study, we showed some alterations/changes in the HM virome composition according to the gestational age, birth weight, and delivery mode. Previous studies on the HM bacterial microbiota have shown differences according to ethnicity, genetic background, maternal body mass index, gestational age, intrapartum antibiotics, delivery mode, infant sex, lactation stage, and method of collection [4,8,11,31,32]. We recently found that the composition of the HM mycobiota varies depending on preterm birth, delivery mode, and birth weight for gestational age, as well as between transient and mature HM [33]. However, the current knowledge of the impact of maternal, infant, and environmental factors on the HM virome is very limited [5]. While we observed some changes according to delivery mode, C-section is not a single factor on the HM virome composition. While nearly all premature babies were born via C-section, the most abundant taxa in transient and mature HM samples were not similar to those in the C-section group, which, in turn, closely resembled those of the normal spontaneous vaginal delivery group. The composition of HM is dynamic, changing with each feeding, diurnally, during lactation, and between mothers. The composition of the body significantly changes during the first month of life in order to meet the needs of the newborn [1]. When compared to milk from full-term mothers, premature birth changes the composition of HM, resulting in substantially higher levels of protein and immunological components [1]. These changes can influence the HM virome composition and vice versa. For this reason, it is difficult to explain HM virome changes with only the delivery mode, and gestational age and factors associated with preterm labor might affect the HM virome composition. In our study, we showed that low birth weight according to gestational age (SGA) also affects the HM virome composition. In the SGA group, the most abundant family was *Herpesviridae* in transient and mature HM samples (44.8% and 48.8, respectively). At the species level, *Roseolovirus*_uc and *Acinetobacter virus 133* were predominant in transient HM samples, and *Human betaherpesvirus 5* was predominant in mature HM samples. The effects of the predominance of *Herpesviridae* in HM samples of the SGA group on the infant gut microbiota composition is unknown. Further studies focusing on mother–SGA infant pairs are required to analyze the relationship between perinatal infections with these viruses and SGA birth or intrauterine growth retardation.

In the first month of life, babies that are predominantly breastfed have been shown to have a significant correlation between the intestinal and HM bacterial microbiota [12,34]. There is no information about an infant’s gut virome or when infant gut viral colonization begins. Some pathogenic viruses have been detected in amniotic fluid and are also thought to be transmitted transplacentally. After delivery, transmission of HM bacteriophages can contribute to the formation of the infant gut microbiome [5,17]. When comparing older children and adults, bacteriophages dominate the early infant virome, leading to a highly dynamic microbiome in early life [5]. Lim et al. [14] studied changes in the gut virome during the first few months of life and found that the gut bacteriophage population structure was predominantly made up of a rich and diverse group of phages, mostly from the *Caudovirales* order. They also found that bacteriophage richness decreased with age after birth, with an increased relative abundance of *Microviridae* at 24 months, resulting in a population with low bacteriophage–high bacterial diversity [9,13,14,35]. Owing to a lack of longitudinal studies, the virome dynamics in HM are unknown. Regarding the changes in the virome from transient milk to mature milk, breastfeeding might play a role in defining the intestinal virome during infancy. In this study, we did not evaluate infants’ stool virome composition. 

The exact origin of the HM virome remains unclear. The bacterial content might be generated from the maternal skin microbiota, entero-mammary pathway, commensal microbiota inhabiting human breast tissue, or the infant’s oral cavity, whereas the origin of the viral content remains unknown [30]. Regardless of the sources of the microbiome composition, infants received these microbial communities regularly during breastfeeding. Human milk microbiota including viruses are among the first microbes to enter the infant’s gastrointestinal tract and play an important role in the development of a healthy microbiota, and in rapid maturation of immunological, metabolic, and neural pathways, and a pioneer role in shaping infant health [3,8,36,37,38]. The effects of the HM virome on infants are not known yet, and further studies are warranted to elucidate its effects on an infant’s gastrointestinal system, immune system, and gut–brain axis. Virome composition changes can either benefit the host or pose a risk of disease. Several studies reported gut virome alterations associated with diseases including infants with diarrhea and malnutrition, and adults with HIV infection, inflammatory bowel disease, colorectal cancer, and type 1 diabetes [5].

Our study has some limitations. A low number of viral reads, unlike bacterial reads, precludes statistical analysis. Most viral sequences are unknown and would not be categorized by a BLAST search, and for this reason, we might have underestimated the viral diversity. We can describe our observations, but the scarcity of data will make intergroup comparisons unreliable. We evaluated the transient and mature HM samples but not the colostrum samples. Detailed metagenomic analysis of human colostrum will complete the trajectory of the HM virome composition. Placenta or amniotic fluid abnormalities, drug treatment other than antibiotics (e.g., acid inhibitors) or vitamin supplementation during lactation might affect the microbiota composition, however, we did not evaluate these factors. Many factors that have the ability to influence the composition of the milk microbiome interact, making it difficult to assess their true impact [39]. We did not collect infants’ stool samples, and also we have no information about the long-term follow-up of the infants. Several studies have confirmed the relationship between maternal dietary intake and the milk microbiota composition. We did not assess maternal dietary intake from the time of birth to the time of milk sampling in our research. Intervention studies need to evaluate the mother’s eating habits throughout both pregnancy and lactation, and food questionnaires to obtain a full understanding of the human milk and the infant gut microbiota are required [2]. The existence of human milk oligosaccharides (HMOs) has been due to the beneficial effects of HM on gut microbiota growth [3,4,40]. The HMO composition was not evaluated in this analysis. There is no detail on the interactions of HMOs with the HM virome. More research is needed to determine how HMOs influence the virome composition of HM.

## 5. Conclusions

In addition to bacteria and fungi, human milk contains eukaryotic viruses and a large number of bacteriophages, which can affect the composition of infants’ gut microbiome [2,16]. Human milk is a complex and dynamic mechanism that allows mothers and babies to communicate with and signal to each other. Every variation of the mother–human milk–infant triad could affect the trajectory of infant development [9]. Our study showed some differences related to preterm birth, birth weight for gestational age, and delivery mode between transient and mature human milk samples, and these factors might affect infants’ intestinal microbiota and also have effects on their health status. The microbial community is dynamic and regulated by cross-kingdom interactions, and any imbalance can impact overall human health [9]. The interconnectivity of the virome with members of the microbiome can have an effect on the health and disease of the host [13,41]. More longitudinal metagenomic, metatranscriptomic, and metabolomic studies involving mother and newborn pairs would help to better define the HM virome and its functional effect on the development of the developing child.

## Figures and Tables

**Table 1:** Maternal age, mode of delivery, gestational age, birth weight, gender, and human milk sampling time of entire study group and subgroups.

	Maternal Age (Years) *	Delivery Mode (SV/CS)	Gestational Age (Weeks) *	Birth Weight (Gram) *	Gender (Boys/Girls)	Transient HM Sampling Time (Days) *	Mature HM Sampling Time (Days) *
Total	30	9/35	38	2840	18/26	8	51.5
(*n* = 44)	(18–44)		(32–41)	(1200–4600)		(7–14)	(45–70)
NS-T	33	8/0	38.5	3225	1/7	12	53.5
(*n* = 8)	(20–41)		(37–40)	(2880–3600)		(8–14)	(47–70)
CS-T	35	0/9	38	3080	5/8	8	55
(*n* = 9)	(25–44)		(37–41)	(2730–3600)		(7–13)	(45–70)
PT	30	0/13	35	2255	3/6	8	50
(*n* = 13)	(18–43)		(32–37)	(1200–2700)		(7–14)	(45–62)
SGA	34	1/6	37	2225	3/4	8	49
(*n* = 7)	(22–38)		(34–41)	(1670–2500)		(7–12)	(45–63)
LGA	30	0/7	39	4120	6/1	8	51
(*n* = 7)	(23–39)		(37–41)	(3505–4600)		(7–13)	(45–63)

* median (minimum–maximum). NS-T: normal spontaneous vaginal delivery-term; CS-T: cesarean delivery-term; PT: premature; SGA: small for gestational age; LGA: large for gestational age; SV: spontaneous vaginally; CS: cesarean delivery.

**Table 2:** Most abundant viruses at species level in transient and mature human milk samples based on delivery type, prematurity, and birth weight for gestational age.

Transient Human Milk (7–15 Days)				Mature Human Milk (45–90 Days)			
Group	Species	n	%	Group	Species	n	%
NS-T (*n* = 432)	*Picornavirinae*	240	55.5	NS-T (*n* = 482)	*Siphoviridae_n__uc*	170	35.2
	Cp1virus__uc	57	13.2		*Siphoviridae_n_n__uc*	69	14.3
	*Staphylococcus phage Andhra*	27	6.25		*Enterococcus phage EFC-1*	30	6.2
	*Staphylococcus phage St 134*	23	5.32		*Picovirinae__uc*	29	6.0
	*Halomonas phage phiHAP-1*	14	3.24		*Human betaherpesvirus 5*	25	5.2
	*Streptococcus virus Cp1*	13	3.0		*Clostridium phage vB_CpeS-CP51*	17	3.5
	*Vibrio phage VP882*	9	2.0		*Brochothrix phage NF5*	16	3.3
	*Human betaherpesvirus 5*	9	2.0		*Sextaecvirus__uc*	14	2.9
CS-T (*n* = 397)	*Sep1virus__uc*	144	36.2	CS-T (*n* = 243)	*Human betaherpesvirus 5*	73	30.0
	*Picovirinae__uc*	51	12.8		*Sep1virus__uc*	67	27.5
	*Siphoviridae_n_n__uc32*	32	8.1		*Siphoviridae_n_n__uc*	19	7.8
	*Streptococcus phage IPP62*	25	6.3		*Myoviridae__uc*	8	3.2
	*Staphylococcus virus SEP1*	20	5.0		*Staphylococcus virus SEP1*	8	3.2
	*Siphoviridae_n__uc*	19	4.8		*Podoviridae__uc*	7	2.9
	*Myoviridae__uc*	13	3.2		*Staphylococcus virus IPLAC1C*	5	2.0
	*Staphylococcus virus IPLAC1C*	11	2.7		*Picovirinae__uc*	5	2.0
PT (*n* = 462)	*Picovirinae__uc*	208	45.0	PT (*n* = 404)	*Human betaherpesvirus 5*	85	21.0
	*Sep1virus__uc*	60	12.9		*Siphoviridae_n__uc*	57	14.1
	*Staphylococcus phage St 134*	34	7.35		*Siphoviridae_n_n__uc*	41	10.1
	*Human betaherpesvirus 5*	26	5.6		*Picovirinae__uc*	19	4.7
	*Siphoviridae_n__uc*	24	5.2		*Lactobacillus phage iLp1308 17*	17	4.2
	*Podoviridae__uc*	13	2.8		*Staphylococcus virus Sextaec*	15	3.7
	*Siphoviridae_n_n__uc*	13	2.8		*Listeria phage B054*	12	2.9
	*Pseudomonas phage phiAH14b*	10	2.1		*Herpesviridae__uc*	12	2.9
SGA (*n* = 176)	*Roseolovirus__uc*	64	36.3	SGA (*n* = 481)	*Human betaherpesvirus 5*	133	27.6
	*Acinetobacter virus 133*	35	19.8		*Siphoviridae_n_n__uc*	98	20.3
	*Siphoviridae_n__uc*	18	10.2		*Roseolovirus__uc*	84	17.4
	*Siphoviridae_n_n__uc*	10	5.7		*Siphoviridae_n__uc*	76	15.8
	*Human betaherpesvirus 6A*	8	4.5		*Streptococcus phage YMC-2011*	15	3.1
	*Human betaherpesvirus 5*	5	2.8		*Streptococcus phage 7201*	10	2.0
	*Serratia phage BF*	4	2.3		*Human betaherpesvirus 6A*	10	2.0
LGA (*n* = 60)	*Siphoviridae_n_n__uc*	16	26.6	LGA (*n* = 158)	*Siphoviridae_n_n__uc*	71	44.9
	*Lactobacillus phage phi jlb1*	7	11.6		*Siphoviridae_n__uc*	18	11.3
	*Human betaherpesvirus 5*	7	11.6		*Sep1virus__uc*	10	6.3
	*Lactobacillus phage phiPYB5*	5	8.3		*Human betaherpesvirus 5*	8	5.0
	*Lactobacillus phage KC5a*	4	6.6		*Streptococcus phage YMC-2011*	5	3.1

NS-T: normal spontaneous vaginal delivery-term; CS-T: cesarean delivery-term; PT: premature; SGA: small for gestational age; LGA: large for gestational age (LGA); n: reads.

## Data Availability

The metagenome datasets from this study are available in the EBI Short Read Archive under the study accession number PRJEB26810 (accession numbers: ERS2488898 and ERS24888985).
